# Radiocarpal Dislocation With Isolated Ulnar Translocation: Case Report and Review of the Literature

**DOI:** 10.1155/cro/8733635

**Published:** 2026-06-27

**Authors:** Maleka Ramji, Mieke Heyns, Emma Yanko, Farrah M. K. Yau

**Affiliations:** ^1^ Section of Plastic Surgery, University of Calgary, Calgary, Alberta, Canada, ucalgary.ca

**Keywords:** carpal bones, joint dislocations, ligamentous injuries, ulna, wrist injuries

## Abstract

Traumatic radiocarpal dislocations are rare injuries. Following high‐energy trauma to the wrist, there should be a high index of suspicion for radiocarpal instability. Global ligamentous disruption of the wrist leads to the carpus translating ulnarly as it follows the inclination of the radius. We present a unique case of ulnar translocation of the carpus in a 33‐year‐old male with a hypoplastic ulna of the injured wrist. Management included open reduction internal fixation and ligamentous repair using a combined volar and dorsal approach to the wrist. The patient was immobilized in a splint for 12 weeks, followed by K‐wire removal and range of motion exercises. At follow‐up 6 months postinjury, the patient had full range of motion, no functional concerns, and no pain in the operative limb. To our knowledge, a pure ulnar translocation radiocarpal dislocation in the setting of a hypoplastic ulna has not been described in the literature.

## 1. Introduction

Radiocarpal dislocations are rare injuries caused by high‐energy trauma. The carpus translates, with or without fracture in the dorsal or volar direction, often followed by ulnar translocation of the carpus given the normal radial inclination of the distal radius [1]. Even rarer are isolated ulnar translocation injuries of the carpus, without dorsal or volar translocation; the incidence of this is not well defined in the literature. Rayhack et al. [2] reported eight cases in the literature of posttraumatic ulnar translocation. All patients had a delay in diagnosis with a median of 7.3 months. Associated injuries included scapholunate dissociation and lunotriquetral dissociation. We present a case of acute radiocarpal translocation, involving a pure ulnar translocation in the setting of a hypoplastic ulna. Urgent operative reduction and fixation were performed. Postoperative outcomes were reviewed at three and 6 months postoperatively.

## 2. Case Presentation

Medical records were reviewed to obtain the patient′s relevant medical history and investigations. Verbal informed consent was obtained from the patient for publication of the article prior to loss to follow‐up. We report a case of a 33‐year‐old male who presented to the emergency department with left wrist pain and swelling after being thrown from a motorcycle during rapid acceleration, sustaining a high‐energy impact to the upper extremity. Past medical history was notable for a left wrist injury managed with splinting alone. Postinjury radiographs demonstrated ulnar translocation of the carpus at the radiocarpal interval without obvious fracture (Figure 1). It was additionally noted that the patient had a hypoplastic distal ulna. Contralateral wrist x‐rays showed a normal appearing ulna. No previous radiographs were available. Computed tomography revealed a small avulsion fracture of the pisiform and triquetrum (Figure 2).

**Figure 1:**
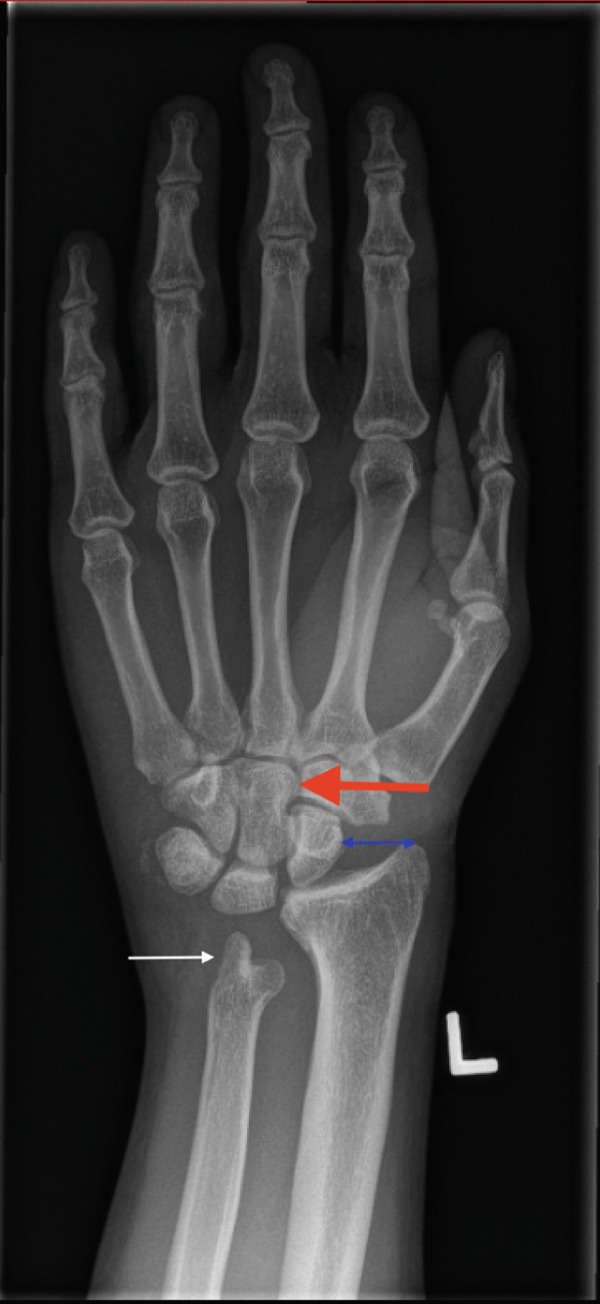
Radiograph of the left hand and wrist, demonstrating ulnar translocation of the carpus (red arrow), relative widening of the radial styloid–scaphoid interval (blue arrow), and a hypoplastic distal ulna (white arrow).

**Figure 2:**
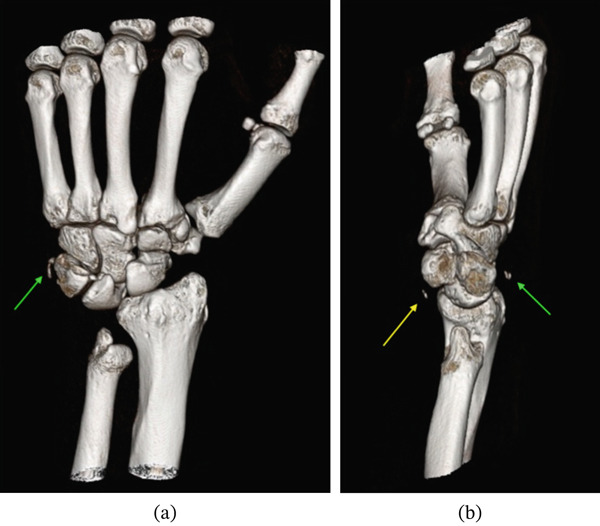
(a,b) CT scan 3‐D reformats of the wrist demonstrating avulsion fractures of the pisiform (yellow arrows) and triquetrum (green arrows).

Closed reduction was unsuccessful in the emergency department. The patient was then taken to the operating room for open reduction and internal fixation. Under tourniquet control, a dorsal incision was made from the mid‐long finger metacarpal to the distal forearm, and the standard dorsal approach to the wrist was used. The capsule was disrupted, thereby not allowing for a capsule‐sparing approach. The dorsal radiocarpal ligament (DRC) was avulsed from its insertion on the triquetrum, with a small avulsion fracture noted. An additional volar incision was made primarily to access the volar wrist capsule; an extended carpal tunnel release was performed concurrently to decompress the median nerve and mitigate the risk of postoperative compression due to anticipated soft‐tissue swelling. The long radiolunate (LRL) and short radiolunate (SRL) ligaments and the volar radioscaphocapitate (RSC) ligament were disrupted. The RSC ligament had a mid‐substance tear. The LRL and SRL were avulsed off their insertion on the lunate.

The reduction of the carpus was confirmed with intraoperative fluoroscopy using axial traction. A total of 0.062‐in Kirshner wires were placed as follows: (1) radius to scaphoid to capitate, (2) radius to lunate, and (3) triquetrum to lunate. Ligament repair was then performed beginning with the volar structures. The LRL and SRL were reattached to the lunate using a suture anchor preloaded with a 3–0 braided polyester suture (Ethibond; Ethicon, Somerville, New Jersey, United States) (Micro‐Mitek; DePuy Synthes, Raynham, Massachusetts, United States). The midsubstance tear of the RSC ligament was repaired using multiple 3–0 braided polyethylene sutures (FiberWire; Arthrex, Naples, Florida, United States). Dorsally, the DRC ligament was repaired with a suture anchor to the dorsal triquetrum. K‐wires were buried and a splint applied.

Postoperative radiographs confirmed hardware placement and adequate reduction of the carpus (Figure 3). The patient was discharged home on postoperative Day 2. At 12 weeks postoperatively, the splint was discontinued and the pins were removed. Radiographs following K‐wire removal confirmed reduction of the carpus (Figure 4). The patient was seen by physiotherapy for range of motion exercises. At the 6‐month follow‐up, the patient had full range of motion and no wrist pain or functional deficits to the limb. The patient has since been lost to follow‐up.

**Figure 3:**
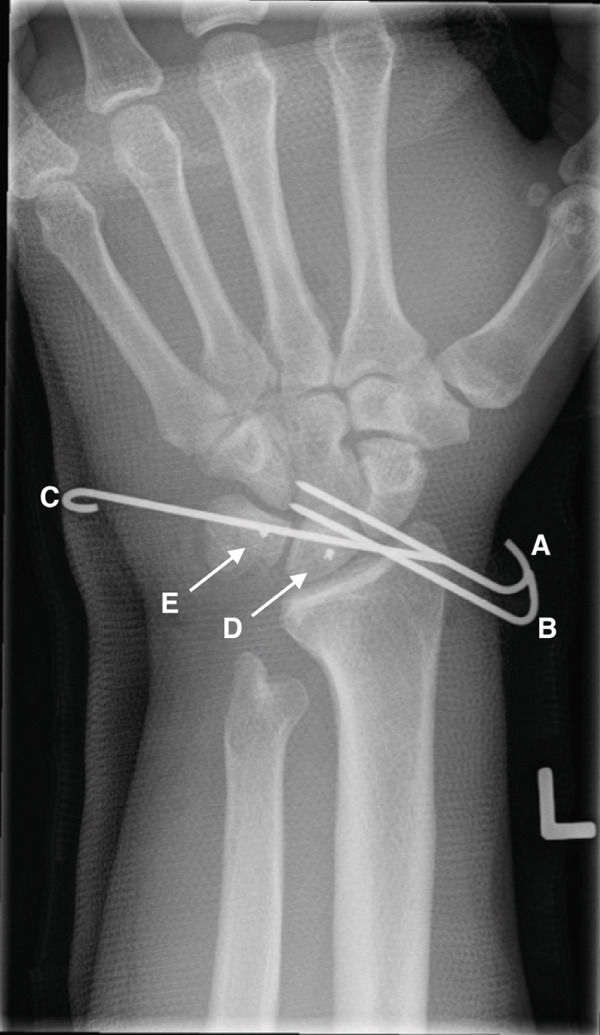
Radiographs of the left hand and wrist immediately postoperatively demonstrating K‐wire fixation and suture anchors for ligament repair. K‐wires, in order of placement: (A) radius to scaphoid to capitate, (B) radius to lunate, and (C) triquetrum to lunate. (D) Micro‐Mitek suture anchor used to reattach the LRL and SRL to the lunate, and (E) Micro‐Mitek suture anchor used to reattach the DRC ligament to the triquetrum.

**Figure 4:**
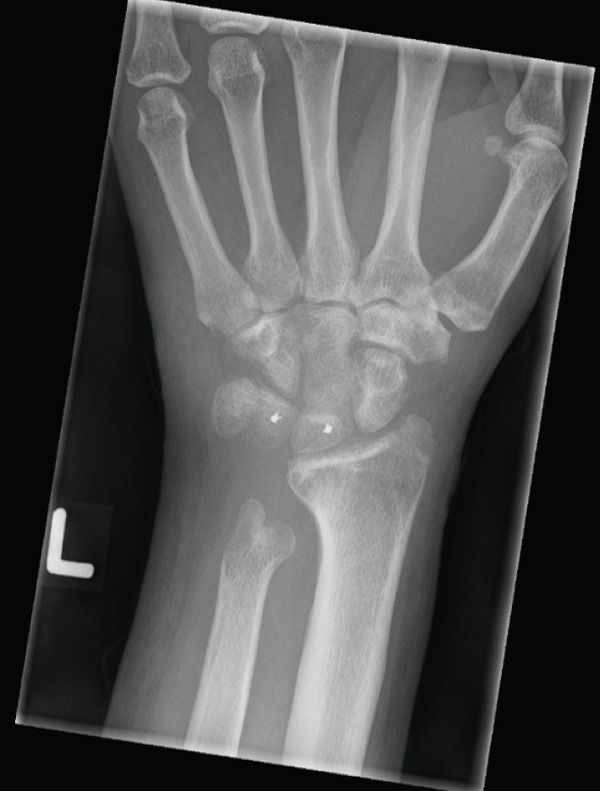
Follow‐up radiograph of the left wrist obtained 3 months postoperatively after hardware removal, demonstrating maintained carpal alignment without recurrent ulnar translocation.

## 3. Discussion

High‐energy wrist injuries encompass a spectrum of rare patterns, including radiocarpal, perilunate, and intracarpal dislocations [3]. Radiocarpal dislocations are uncommon, accounting for up to 3% of distal radius trauma cases [4]. Isolated ulnar translocation of the carpus is even rarer, with dorsal and volar translocations more commonly reported [5].

A retrospective review of 27 patients with radiocarpal dislocations described a range of treatment strategies, including closed reduction and casting, percutaneous K‐wire fixation, external fixation, and open reduction with ligament repair [6]. Reported complications included posttraumatic arthritis, recurrent subluxation, and decreased wrist range of motion. Notably, pure ligamentous radiocarpal dislocations were associated with a higher risk of secondary ulnar translocation compared with those with an associated radial styloid fracture.

Taleisnik et al. [7] described two patterns of ulnar translocation at the radiocarpal joint: Type I, involving ulnar translocation of the entire carpus, as seen in our case; and Type II, characterized by complete scapholunate ligament disruption with ulnar translocation of the carpus while the scaphoid remains within the scaphoid fossa. To our knowledge, isolated ulnar translocation in the setting of a hypoplastic ulna has not been previously reported.

Under normal anatomical conditions, the ulnar head and triangular fibrocartilage complex (TFCC) provide an important ulnar‐sided buttress to the carpus, resisting ulnar translocation. Disruption or deficiency of this restraint has been implicated in traumatic ulnar translocation [1]. Additionally, ulnar‐negative variance has been shown to increase susceptibility to posttraumatic ulnar translocation [2]. In the present case, the hypoplastic ulna likely resulted in a relative deficiency of ulnar support, predisposing the wrist to isolated ulnar translocation.

In the largest case series on this topic, Berschback et al. [5] reported nine patients (10 wrists) with traumatic ulnar translocation, all treated surgically within 2 months of injury. Procedures included various combinations of radiocarpal ligament repair, radiocarpal pinning, and fracture fixation depending on the injury pattern. At a mean follow‐up of 6.5 years, functional outcomes were favorable, although radiographic translocation persisted in nine of ten wrists and arthritis developed in six wrists. Ulnar minus variance was present in nine cases, suggesting that the absence of an ulnar‐sided bony buttress may predispose to ulnar translocation.

An earlier series by Rayhack et al. [2] described eight patients with posttraumatic ulnar translocation. Seven underwent ligamentous repair, one required radiolunate arthrodesis, and three ultimately progressed to total wrist arthrodesis due to persistent pain. Radiographic recurrence of ulnar shift was observed in several cases, consistent with patterns seen in dorsal or volar radiocarpal instability.

More recently, Brown et al. [8] reported a case of traumatic radiocarpal translocation managed with combined repair of the volar and dorsal ligamentous structures, further supporting restoration of radiocarpal stability in acute ligamentous injuries.

In contrast, Nasra et al. [9] reported nonoperative management of a chronically missed ulnar translocation in an elderly patient, highlighting that treatment decisions should be individualized based on timing of presentation, patient factors, symptoms, and functional demands.

Similarly, Arslan et al. [10] described a patient treated with external fixation for 6 weeks who developed recurrent ulnar translocation and persistent pain at 1 year. In another report, two patients treated with ligamentous repair and K‐wire fixation demonstrated mild reductions in range of motion and grip strength, with radiographic recurrence of ulnar drift at 1‐year follow‐up [11].

There remains no consensus regarding optimal management of these injuries, and patients should be counseled on the risk of recurrent translocation. In response to this challenge, Maschke et al. [12] proposed a cadaveric reconstruction using a distally based brachioradialis tendon passed through the capitate and anchored to the distal radius to recreate the RSC ligament; however, clinical application of this technique has not been reported.

In summary, posttraumatic ulnar translocation of the carpus is a rare injury with limited published data. No single treatment strategy has demonstrated clear superiority in maintaining carpal alignment or preventing recurrent ulnar translocation over time.

## 4. Conclusion

Traumatic radiocarpal dislocations are rare, with isolated ulnar translocation representing an even less common injury pattern. This case highlights a previously undescribed association between a hypoplastic ulna and isolated ulnar translocation, suggesting a potential anatomical predisposition due to reduced ulnar‐sided restraint. Successful management was achieved with open reduction and internal fixation using combined volar and dorsal approaches, resulting in a pain‐free wrist with full range of motion at 6 months. Given the rarity of this condition, continued reporting of similar cases is important to guide future management strategies.

## Author Contributions

Conceptualization: M.R. and F.M.K.Y.; methodology: M.R. and F.M.K.Y.; data curation: M.R. and F.M.K.Y.; writing—original draft preparation: M.H., M.R., and F.M.K.Y.; writing—review and editing: M.H., M.R., E.Y., and F.M.K.Y.; supervision: M.R. and F.M.K.Y.; project administration, M.H., M.R., E.Y. and F.M.K.Y.

## Funding

No funding was received for this manuscript.

## Disclosure

All authors have read and agreed to the published version of the manuscript.

## Ethics Statement

Our institution does not require ethical approval for reporting individual cases.

## Consent

Verbal informed consent was obtained from the patient for publication of their medical information and images. Written consent could not be obtained from the patient as the patient was lost to follow‐up.

## Conflicts of Interest

The authors have nothing to report.

## Data Availability

The authors have nothing to report.
